# Hospital and Institutional News

**Published:** 1916-02-12

**Authors:** 


### HOSPITAL AND INSTITUTIONAL NEWS.

### THE FLORENCE NIGHTINGALE MEMORIAL.

As we state in our leading article, Her Majesty the Queen will attend at St. Paul's Cathedral the unveiling of the above memorial in the Crypt at 2.15 P.M. on Monday, the 14th instant. The memorial has been designed by Mr. Arthur G. Walker, the eminent sculptor. The Archbishop of Canterbury will be present, and a service will be held under the Dome after the unveiling, seats being reserved for the committee and nurses who may have previously obtained tickets by application to the Hon. Secretary, Florence Nightingale Memorial Committee, St. Thomas's Hospital, Albert Embankment, S.E.

### THE LATE MR. WALTER G. CARNT.

It is with deep sorrow that we have to record the death, on Friday, the 4th instant, of Mr. Walter G. Carnt, late superintendent and secretary of the Manchester Royal Infirmary. He passed away quite peacefully at Coombe Leigh, Ryde, and the sympathy of everyone who knew him will go out to his widow and family. We have had the most intimate and continued association with Mr. Carnt for many years, and have always found him one of the ablest, most devoted and generous workers in the hospital field. He was untiring in devotion to duty, ever willing to lend a helping hand to co-workers and to all who were genuinely interested in the welfare and uplifting of the voluntary hospital system. He belonged to the rare type of man who has the courage to think out for himself and enforce sound principles of administration, and never to be satisfied with anything less than the best attainable. It has been our privilege to come into continuous communication with hospital workers throughout the world. It is not too much to say that few men have exhibited an equally generous readiness to communicate information, to supply facts, or to co-operate in the progressive development of everything which contributes to the maintenance and progress of the highest standard of hospital administration and hospital work. We are grateful to remember that Mr. Carnt was permitted to fulfil his wish to continue his work to the very last moment of a busy and useful life. He was buried on the 8th instant at Whippingham, Isle of Wight. A full biographical notice of Mr. W. G. Carnt will be found in The Hospital of September 4, 1915, p. 469 *et seq.*

### THE ROYAL RED CROSS.

The Royal Red Cross (first class) has been awarded, in recognition of valuable services rendered in the organisation and training of Voluntary Aid Detachments in connection with the war, to Miss S. A. Swift, matron-in-chief of the Joint War Committee, her matrons Mrs. H. Corner and Miss E. M. Roberts, and to the Viscountess Esher, Lady B. C. Oliver, and Mrs. K. Furse, representing Voluntary Aid Detachments. The King has also sanctioned the appointment of Miss S. A. Swift to be a Lady of Grace of the Order of St. John of Jerusalem in England, and Miss C. H. Keer, Mrs. Edred Corner, and Miss E. M. Roberts to be Honorary Serving Sisters of the same Order. We offer our congratulations to all these ladies upon the honours conferred upon them. Miss Swift has done yeoman service since the beginning of the war, and the organising ability and sound judgment she has exhibited prove her to be one of the ablest matrons and nursing authorities of modern times. She is a worker and not a talker. The results she has achieved, not only in her present high position but in connection with the Royal National Pension Fund for Nurses and for Guy's Hospital, to mention only two of several fields of labour to which she has devoted her energies, cannot be too highly praised. We wish her long life, good health, and sustained energy to carry through to completion and success, all the many excellent movements of which she is the centre and controlling spirit.

### SOLDIERS AND CIVIL HOSPITALS.

In spite of two semi-official explanations in the *Times* of the attitude at the moment of the Army medical authorities to the civil hospitals which are treating soldier patients, there is much uncertainty and some consternation among the managers of the institutions as to exactly how and where they stand. This state of things is attributable partly to the lull on the Western battle-front, and to the excellent health of the troops there, neither of which factors is a reproach to the Army Medical Service; and partly, we think, to some lack of consideration at the War Office, where the peculiar difficulties of the voluntary charities do not seem to be quite clearly understood. The position, so far as we can learn, is briefly this. Great preparations were made last summer, and rightly so, for the reception of large numbers of casualties; and thousands of beds were prepared, many of them in civilian hospitals, whose offers the War Office accepted thankfully. Just now, there are not nearly enough patients to fill these beds. It struck the War Office authorities as economical to order that the purely military hospitals, directly managed by them, should be kept full, or as nearly so as possible; while the loaned beds in the civilian hospitals should be held empty for future emergencies. Notice was accordingly given of this intention; and in some cases the hope was expressed that the empty beds would be available at call when more active hostilities are resumed.

### WHERE THE SHOE PINCHES.

This suggestion, not unnaturally, put the managers of many institutions in a difficult position. They saw themselves faced with the loss of present revenue from the War Office, which is on a capitation basis per occupied bed; and with one of two further consequences, each detrimental to important interests. If the beds are to be kept permanently vacant until after the next big action, some of the sick civilian poor may have to be refused relief which those empty beds could afford. If the beds are thrown open to such patients, the wards cannot be emptied at once when the need comes for the soldiers, for it takes at least four or five weeks to close down a ward when once it is full of sick people. Hence the managers felt that they must either fail in their duty to civilians, or else be unpatriotic. This dilemma does not seem to have been foreseen at the War Office, or not to have been sufficiently considered. Probably by this time it has been comprehended, and we hope that it will be sympathetically taken into account. At any rate, we are grateful to Sir Alfred Keogh for the latest order, which supersedes those of a few days previously, and is much less irksome to hospital managers who have lent their beds to the Army medical authorities.

### THE DRAMA AND ST. THOMAS'S HOSPITAL.

The *St. Thomas's Hospital Gazette* draws attention to the fact that two plays by St. Thomas's men have been running in London ("The Parish Pump," by Mr. F. G. Layton (now withdrawn), and "Please Help Emily," by Mr. H. M. Harwood), whilst a third, from the most distinguished of all living medical playwrights, Mr. Somerset Maugham, made its first appearance this week. It must be a record for the *alumni* of one medical school to have two plays before the London public at one and the same time. Is it just coincidence? Or is there something in the air near Westminster Bridge that conduces to dramatic authorship? Or perhaps in the training at that celebrated institution? At any rate, it is undeniable that St. Thomas's Hospital leads the way, be the explanation what it may.

### AN OPPONENT OF THE ASYLUM ATMOSPHERE.

With the appointment of Dr. William Henry Coupland to the post of medical superintendent of the Royal Albert Institution, Lancaster, the institution possesses in its new chief not only its former senior assistant medical officer, but also an official who has been connected with it for nineteen years. Educated at Edinburgh University, Dr. Coupland's appointments have included resident posts at North Staffordshire Infirmary, Stoke-on-Trent, and also at the Stoke Union Hospital. It is interesting to add that Dr. Coupland has exhibited various activities within and without the pale of mental hospital work. He has engaged in the study of the problems associated with recent legislation affecting feeble-minded and mentally defective persons; he has been a busy publicist; and his general interest in science is apparent from the fact that he helped to found the Lancaster Astronomical and Scientific Association, of which he is now the president. The Lancaster Medical Book Club also owns him as its chief. It is always pleasant to record instances of men engaged in mental hospital life who have refused to fall into the rut of routine and despairing self-sufficiency; who do not lose their early interest in science in spite of little or no encouragement, but maintain their energies in spite of the premium which is placed on inertia by a branch of institutional life. In this branch the prizes are very few and the routine is for the most part monotonous and somewhat cut off from the beneficial stimulus of social and professional life.

### A BIRMINGHAM HOSPITAL UNIT FOR THE FRONT.

The War Office have given authority to Lieutenant-Colonel Gilbert Barling to raise in the Birmingham district a hospital unit and medical staff for service abroad, probably in the East. This hospital will contain 1,040 beds with a staff of thirty doctors, including the commanding officer and Registrar. Nearly the whole of these have been secured. Three members belonging to the staff of the General Hospital have been spared by the committee, and most of the other gentlemen are in practice in Birmingham. Unfortunately, circumstances will prevent Lieutenant-Colonel Barling from accompanying the unit. Owing to the fact that the Birmingham University (of which he is Vice-Chancellor) is surrounded by not a few difficulties at the present time, and to Lieutenant-Colonel Barling's many other important duties in the City, it is impossible for him to go abroad.

### THE ORDER OF ST. JOHN.

Several new promotions and appointments have been sanctioned by the King in the Order of the Hospital of St. John of Jerusalem in England. There is but one medical man in the list; Inspector-General Ninnis, R.N., is promoted Knight of Justice from Knight of Grace. On this occasion a good many ladies receive recognition: the most interesting, perhaps, of these recipients is the lady officially described as Helen Porter, but better known as Mme. Melba. Of seven new Knights of Grace, there are none with institutional connections, so far as we are aware: apparently only one of them has any previous connection with the Order. A few more "commissions from the ranks" would probably strengthen the position of this Order much better than the present system of bestowing its knighthoods. Have financial considerations ever anything to do with the bestowal of this Order? What fees have to be paid?

### THE LAST OF THE INDIAN HOSPITAL.

With the past week the latest and most anxious phase through which Dome and Pavilion at Brighton have passed came to an end with the final departure of the Indian wounded. The occasion was fitly marked by throwing open the interior to the public at a nominal charge, whereby the inhabitants of Brighton had a unique opportunity of seeing at first-hand the hospital as the Indians left it—except, no doubt, for a general tidying-up—and at the same time a substantial sum was raised for the benefit of the Mayor of Brighton's "War Charities" fund. It is just over a year since the Royal Pavilion was opened as a hospital for the wounded, and the period from December 14, 1914, to the end of January 1916 has enriched the romantic associations of the curious edifice (it can hardly be called a building) in a most remarkable fashion. Structural alterations have been limited to the "offices"; the mural decorations have been encased, like the carvings of St. Mark's, Venice; and the corridor remains unchanged. On the other hand, the associations of the place have been multiplied indefinitely. And so also, for the time being, at any rate, have been the sights. These now include the site on which the praying-tent was erected; the spot on the lawn where the King presented the V.C. and other decorations to the Indian soldiers; the huts on the lawn; and, more especially, the fine state apartments as they were left by the Indians: the rows of beds and signs and symbols, hygienic or religious, by which the rules of caste or ritual observances were carried out.

### A MODEL FOR HOSPITAL SPEECHES.

We have often pointed out that a very good way to make the annual meeting interesting is to provide good speakers—men, that is to say, who can communicate to others the interest in hospital work which is taken by those who are actively engaged upon it. But the hospital is a lucky institution which has to go no further afield for such a speaker than its own treasurer or chairman. Nevertheless such hospitals exist, and a capital instance is provided by the Radcliffe Infirmary, Oxford, at the recent annual meeting of which its able and hard-working honorary treasurer, the Rev. G. B. Cronshaw, delivered a speech, reviewing the year's work, which was a model of what such an address ought to be. (It can be referred to at length in the files of the *Oxford Times*.) First he gave a picture of the part which his institution plays in the life of the county and city; of the common centre which it is becoming of "every effort which was put forward either by public authorities or private associations for medical aid." Therefore he described how the medical officers of health for both the city and the county had been added to the staff, and how week by week during the past year groups of medical practitioners of the district had gathered for clinical discussions with members of the staff. Mr. Cronshaw then gave a most interesting description of the way in which the staff have done a double share of work, at the base and at the infirmary, and of the honours which have come to some of them, including the matron. The work of the new special departments was then described, and the growth of the Thursday clinics for mothers, a recent activity which he prophesied would have a large future before it. The financial statement, with the wonderful part played by the number of gifts in kind, was then explained, and the speech ended with acknowledgments, unmistakably sincere, of the work done by every employee in the institution. The material of the address, it will be seen, was not unique; but the spirit in which the speech was given made every statement alive with interest; and the fact that it can produce a similar impression in the reading is the best of all tributes to Mr. Cronshaw's own enthusiasm. No wonder a speaker told him that a speech on behalf of the Radcliffe Infirmary always drew an attentive audience. Enthusiasm is infectious, when it is the real thing.

### FORMER SECRETARIES ON HOSPITAL BOARDS.

It used sometimes to be debated, and doubtless is so still, whether ex-hospital secretaries make good members of hospital boards when they have retired themselves from active work. The real answer to the question, however, depends on whether the questioner contemplates the ex-secretary as a member of the board of the hospital which he used to administer, or whether he is to give the benefit of his experience to those elsewhere who have never exercised authority over him. Usually, of course, secretaries who become members of hospital boards on retirement are appointed, like members of the consulting staff, to their own institution, and where a compliment is merely intended no debatable matter need arise. But whether a retired secretary is well advised to put in regular appearances at the board meetings which it used to be his duty to attend is far more doubtful. The particular routine of the institution he must know well, if not too well indeed; but is this knowledge of detail and personalities likely to be helpful to him in taking the more external independent view which should be that of a useful member of a board of management? In the rare cases where two ex-secretaries have become members of their hospital board, it is probable that the advantages are doubled and the drawbacks are halved. This view, at least, is put forward by the committee of the Throat and Ear Hospital, Brighton, who congratulate themselves on the advantage of having "two former secretaries to serve on the committee." To explain how this came about it is very necessary to remind our readers that when Mr. E. Pilbeam retired not long ago, Mr. R. B. Phillips was appointed. But when pressure of other work led to Mr. Phillips' retirement he consented to serve on the committee, and Mr. Holmes was appointed in his room last November. The somewhat unusual situation has been brought about by the quick change of officers; but though this affects the practical bearing of the situation, the principle should not be lost sight of.

### THE "SOUTHERN CROSS" AGAIN.

The second number of the *Southern Cross*, the journal of the 1st Southern General Hospital, Birmingham, is quite as good as the first, the contents of which we described to our readers on its appearance. It is pleasant to note that the editor seems to have got into his stride, and throughout the magazine an air of flippancy is agreeably maintained. For flippancy has its virtues; and the instinct for reverence itself could not exist were there not an instinct for irreverence to keep the former from making itself ridiculous. The illustrations, perhaps, are less uniformly good this time than the sketches and articles; but the lively spirit of the whole, which is not dependent on merely silly jokes, speaks well for its future issues.

### CONSULTING SURGEONS FOR LEEDS WORKHOUSE INFIRMARY.

The example set by Sheffield is to be followed by the Leeds Guardians, who have just decided to appoint consulting surgeons at their workhouse infirmary in Beckett Street. The result of the scheme is that pauper patients who require major surgery will have it performed by specialists, paid out of the rates, instead of by the ordinary staff. Not only the usual emergencies of general surgery, but also those of ophthalmic, naso-pharyngeal, and aural surgery will be thus entrusted to experts, as well as obstetric cases of unusual difficulty. It is undoubtedly proper that in these days of specialisation cases which are outside the ordinary run of the general surgeon should be submitted to those who have made a study of the particular special branch of surgery concerned, and the Leeds Guardians are taking a step which has proved satisfactory at Sheffield. There is, however, one conceivable drawback to their policy, and that is the prospect that the Poor-Law medical service may thereby be made even less attractive than it is now, and that, in consequence of these appointments, there may be a falling off in the quality of those who seek appointments under Boards of Guardians which follow the example of Sheffield and Leeds.

### THE NOTIFICATION OF PREGNANCY.

The Supplement to the Forty-fourth Annual Report of the Local Government Board (for 1914-15), containing the report of the medical officer, contains some valuable suggestions for the management and organisation of ante-natal care centres. It is especially noted that a system of notification of pregnancy is not essential, and that, when such a system is introduced, express consent of the expectant mother should be obtained in every case. This important proviso repeats the advice given in the Special Supplement (Cd. 8,085) on Maternal Mortality, to which allusion was made in these columns in the issue of December 4 (p. 202). The impression then given that this valuable safeguard has been tardily recognised is now proved erroneous, we are glad to find, for the latest Supplement (Cd. 8,153) repeats the language of the December issue in identical words.

### A PATIENT'S RECEPTION AT LISCARD HOSPITAL.

Mr. G. C. Bate, the Wallasey District Coroner, and a jury have had to investigate a painful case. The facts may be briefly stated. An elderly woman, who lived at Seacombe, becoming unconscious after a fall, was lately taken to the Victoria Central Hospital, Liscard. Here she was seen by a nurse, who, after being told by the son of the deceased woman that his mother had a wound in the arm and was unconscious, declared, after having informed the hospital doctor, that the hospital was full and that it was no case for the institution. After removal from the hospital the woman was examined by Dr. F. W. Inman, who found her "deeply unconscious," with paralysis of the right arm and right leg. A week later she died. In answer to the Coroner, the nurse stated that she did not fully examine the case, but reported the symptoms to the doctor, who was in bed, and that "it was not her duty to examine patients. She only looked for symptoms." The doctor ordered the woman to be sent to the workhouse infirmary, but the relieving officer would not agree. The relieving officer said that the nurse rang him up, and was anxious for him to admit the patient. In answer to his suggestion that she should wait till he could see her, the nurse replied that the woman could not be kept in the hospital. He went therefore to the woman's home, where he found the patient's relatives indignant at the treatment received at the hospital. They refused to hear of her admission to the infirmary, and called in Dr. Inman.

### THE CORONER'S IRONY.

The Coroner said that they could not help feeling that the woman had not been treated in a way the hospital committee would approve. On this the chairman of the hospital committee, Mr. C. R. B. M'Gilchrist, declared that the management were very jealous of the hospital's reputation; that the doctors were run off their feet; that the nurses were highly trained; that every bed was occupied; and, finally, that to have kept the woman on a temporary stretcher would not have conduced to her comfort. In the report of Mr. M'Gilchrist's speech, which lies before us, there is no word of regret, and, in face of this, our readers will appreciate the full irony of the Coroner's quiet words:—"We understand now that whatever occurred when the woman was admitted is approved by the hospital committee." We fear that a very bad impression must follow Mr. M'Gilchrist's speech. Already, in fact, an emphatic protest has been entered by a member of the Wallasey Council, which contributes £300 a year to the hospital's funds.

### ANOTHER RED CROSS SALE.

Last spring, it will be remembered, an extremely successful sale was held on behalf of the Red Cross Society and the Order of St. John, which realised nearly £50,000. This year it has been decided to repeat the experiment, and it is sincerely to be hoped that an equal, or even greater, degree of success will attend the sale. The goods to be sold include works of art, jewels, antique clocks, miniatures, old silver, china, English and French prints, books and manuscripts, tapestries, snuff-boxes, medals, historical relics, pictures, drawings, and small pieces of antique furniture. A strong committee is responsible for collecting the objects of art for sale; and there are sub-committees for different branches such as books and manuscripts; furniture and china; and old silver. This is a wise arrangement, inasmuch as no one can be sufficiently expert in all these subjects to appraise properly the articles sent in. Messrs. Christie, Manson, and Woods have generously consented to conduct the sale free of all expense. The committee, in appealing for gifts, reserves the right to omit from the sale any article deemed unsuitable, or to offer it for sale at some subsequent auction.

### THE ENGLISH IN ITALY.

THE *Tribuna* declares that the British colony at Florence, which has long been anxious to show its sympathy with the Italians in their alliance with England, on the suggestion of an English lady, the Marchesa Ginevra Niccolini, has made provision for the reception for special treatment of wounded soldiers after their discharge from other hospitals, when still suffering from partial paralysis or crippled condition of the limbs calling for massage, baths, and electrical treatment. The Marchesa Niccolini, with her friends, consulted Professor Burci, who obtained for them the handing over of the establishment of baths in the Via Bonifazio Lupi, Florence, the property of the Bonifazio Hospital. This establishment was equipped with a complete plant for medicinal baths and electrical treatment. A committee was appointed, the necessary funds have been raised, and the baths establishment referred to has been equipped and furnished for the accommodation of forty patients, being Italian soldiers. It appears that each "bathing cabin" has been furnished as a separate compartment for the accommodation of one patient, and everything is being done to secure the comfort and happiness of the inmates. Good results have already been obtained. We are glad to record this practical example of British sympathy with the Italian soldiers and people.

### TRENCH FOOT AT THE FRONT.

A DISTINCT sense of disappointment was experienced both by the medical profession and the public at home, and by the Royal Army Medical Corps authorities in France, when it was announced that with the advent of a spell of very severe weather last November a good many cases of "trench foot" were notified. It was thought that the elaborate precautions taken would have prevented, altogether or very largely, that fertile source of military wastage. Since the cold snap of November the winter has been exceedingly mild, though unusually wet, resembling in this latter respect the winter of 1914-15. No official statement has been made lately; but it is gratifying that the general opinion of medical officers in base hospitals, both in England and in France, seems to be that the cases this year do not number more than a quarter, even if as much as that, of those under treatment at the corresponding time a year ago. Doubtless the mildness of the weather is in part responsible; but even more credit must be ascribed to the precautionary remedies advised by the Army medical authorities and to the way in which the combatant commanders and the regimental medical officers have supervised their application by those under their charge.

### THE ROYAL SALOP INFIRMARY.

THE annual report of this institution for the year ending June 30, 1915, shows evidences of an excellent spirit of enterprise in facing the difficult conditions imposed by the strain of war upon so many voluntary charities. It is true that, even after taking in legacies, there was a deficit of £1,650 on the year's working, instead of a surplus of £2,800 as in the previous twelve months (which was a bumper year for legacies). But in some other respects there is progress to note which is quite satisfactory. Subscriptions, donations, and collections all showed very fair increases. Further, the anniversary meeting brought in a record sum, nearly three times as great as the average thus realised in the last few years; the credit for this is ascribed to Mr. A. C. McCorquodale, the treasurer, who has evidently been a tower of strength to the infirmary. The Ladies' Auxiliary Committee completed its first year of work, with very beneficial results. Lady Harlech is to be congratulated on the progress already made, which is doubtless only a foretaste of what her organisation will in time attain to. Among other changes the constitution of a Staff Appointments Board (of forty-one members) may be noted as a step in advance; and another new departure is the institution of a professional paid audit in place of the honorary services so long rendered by Messrs. Sandford and Salt, whose valuable help was acknowledged in a special resolution of the Board.

### THE SUPPLY OF PETROL FOR DOCTORS' CARS.

AT a meeting of the Insurance Committee for the Borough of Leicester, presided over by Mr. H. Williams, a medical man called attention to the local restrictions which he said were being imposed upon the supply of petrol to doctors. He stated that the biggest suppliers of petrol in the neighbourhood had refused to supply private consumers, including medical men. Within certain limits petrol would be supplied for commercial purposes, but doctors were not included in this proviso. He pointed out that at a time of much work like the present, medical men would not be able to cover the distances which were now expected of them, with the result that matters might become serious for their patients. The decision was characterised as absurd, and the clerk was instructed to inform the Insurance Commissioners of the circumstances. As things are it certainly seems absurd that medical men should be victimised in this fashion, and the sooner the plan is given up the better.

### THE SHORTAGE OF DOCTORS.

ON the ground that there are only two doctors left to attend to 1,000 sick patients and 400 wounded soldiers, the Manchester Board of Guardians has refused, after a long and sharp discussion, to release Dr. Marsden, medical officer at their Crescent Road Hospital, in order that he may proceed on foreign service with the R.A.M.C., in which he holds a commission. It was stated that when the other doctors left the two remaining made a joint application for an advance of their salaries from seven to ten guineas a week, adding that if the application was not granted the Board would lose their services. The result was that the Guardians came to an arrangement whereby the salaries were increased to nine guineas a week. The dearth of medical men was so serious that the Board, it was stated, were prepared to appoint women doctors.

### PUBLIC PHARMACISTS' NEW CHAIRMAN.

Mr. Richard Welford, the newly elected chairman of the Public Pharmacists' and Dispensers' Association, has been an active worker in the cause of public pharmacy for many years, and has already acted as chairman for the year 1905. Mr. Welford, who is pharmacist in charge of the dispensary and pharmaceutical laboratory at Colney Hatch Mental Hospital, is a thoroughly practical pharmacist and a firm believer in manufacturing, as far as possible, all the galenicals and similar preparations required in large institutions, not only from the standpoint of economy but also on the score of obtaining reliable pharmaceutical products. At the present time some added manufacturing plant is being laid down for the manufacture of tablets, the preparation of distilled water, etc., which will place Colney Hatch Dispensary in a position comparing favourably with that of a large general hospital. Many matters affecting the position and status of institutional pharmacists will require their attention in the near future, and the Association is fortunate in having such a competent leader to direct its energies.

### DRUG CONTRACT FOR A MILITARY HOSPITAL.

The great advances which have taken place in the prices of drugs, together with the extreme demands made by the military authorities for rare drugs, difficult to obtain, have led to a friendly dispute between the Southwark Board of Guardians and their drug contractors, who have supplied them with drugs for many years. The Southwark Infirmary was taken over by the War Council as a military hospital, and their contractors, who had made provision for a normal supply of certain drugs, found that under the new administration they were being called upon to supply drugs in larger quantities than heretofore. This particularly referred to those of a more expensive character, which in the old days, when the infirmary was completely under the control of the Guardians, had been restricted as much as possible by the medical staff. The contractors desired to know how they stood, as they were losing heavily on their contract under the new conditions. The Guardians realised that they were bound to supply whatever the military medical staff required, irrespective of cost, whilst at the same time they felt they could not allow an old and valued firm of contractors to suffer, though by their contract the firm was bound to supply whatever was required. The Guardians, under the circumstances, decided that their drug contractors should continue to supply drugs to the military hospital at the current market prices, and not at those scheduled in their tender. As for the other institutions under the Guardians' control, the firm would be expected to continue their supplies at contract rates, subject to any abnormal rise in prices, which would be considered at the end of the quarter.

### THIS WEEK'S DRUG MARKET.

Although since the beginning of the year there has been no remarkable advance in the prices of any of the synthetic drugs, there are at present no signs of any impending reductions in value; and, with the exception of hexamine, values are well maintained. The policy which prudent buyers are adopting, however, is to purchase such drugs not in large quantities, but to buy sufficient to satisfy the requirements of the near future. Bromides are somewhat more freely available, but there appears to be no immediate probability of any reduction in price. Further business has been done in quinine at increased rates, and it seems inevitable that the value of this drug must remain, for the period of the war, on a higher basis. Potassium permanganate is scarcer than ever, and is steadily advancing in price. Other drugs which are dearer are scammony resin, strychnine, chrysarobin, cream of tartar, nux vomica, citric acid, tartarated iron, balsam copaiba, and Russian cantharides. On the other hand, there is a lower tendency in the prices of chloral hydrate, balsam tolu, and ergot of rye, while citrates are slightly cheaper, which is rather surprising in view of the fact that citric acid is dearer. For the present the advanced price of cocaine is maintained. The market for cod-liver oil is attracting attention, and should be followed closely by intending buyers; the Norwegian fishing has begun, and while reports indicate that the fish caught up to the present show a good yield of oil, no reliable opinion can be based on this fact, because there is a shortage of fishermen, so many of whom have joined ocean steamers as sailors. In the meantime, however, the value of last season's oil is not quite so firmly maintained; most buyers are inclined to wait a little before placing their orders.

